# 659. Correlating Nurse Employee Engagement with Professional Development, Governance, and Communication on a Pediatric Burn Unit

**DOI:** 10.1093/jbcr/irag033.496

**Published:** 2026-04-06

**Authors:** Lisa M Shostrand, Emily Dever

**Affiliations:** Riley Hospital for Children at Indiana University Health, Indianapolis, IN; Riley Hospital for Children at Indiana University Health, Indianapolis, IN

## Abstract

**Introduction:**

Supporting pediatric burn nursing staff through targeted professional development, governance and communication can influence employee engagement by empowering nurses to feel more involved in their work environment. Professional development initiatives encourage skill and career advancement, while practice governance allows participation in decision-making processes and autonomy. Communication, especially through leadership rounding, promotes trust and team cohesion. These strategies created a more positive workplace culture, which was reflected in higher employee engagement as nursing reported greater satisfaction and commitment to their roles.

**Methods:**

A new model was introduced to the Pediatric Burn Critical Care Unit, including professional development, nursing professional governance, and increased leadership communication in 3rd quarter 2023. Professional development included self-selection of projects, goals, and committee involvement congruent with team members’ interests and organizational vision. Professional governance council experienced growth in clinical practice issues discussions, resulting in the genesis of burn clinical advisor roles. Multi-modal communication included daily leadership rounding and the promotion of the unit’s daily management systems board, which included harm metrics, engagement scores, patient satisfaction data, quality improvement projects, and burn clinic metrics. Employee engagement scores were tracked over a four-year period between 2022 and 2025, the pre- and post-model implementation period.

**Results:**

Full employee engagement data was tracked during the 3rd quarter annually from 2022 to 2025. Nurses were asked to complete a survey with separate scoring for thirteen separate questions in addition to an overall mean. Questions were based on a Likert scale and were reflective of the following categories: nursing quality care fundamentals, leadership access and responsiveness, autonomy, interprofessional relationship, teamwork, professional development, and resource adequacy. A positive increase in scoring occurred in eleven out of thirteen questions, with an overall mean increased from 3.09 in 2022 to 4.02 in 2025.

**Conclusions:**

Engagement scores advances were directly correlated with upward trends in development, teamwork and autonomy. Additional opportunities for improvement and processes continue to be developed based upon survey results and continuous dialogue amongst staff.

**Applicability of Research to Practice:**

Offering collegial support to aid in practice growth, professional governance to promote autonomy, and enhanced leadership communication to build foundational trust may lend to increased commitment to the field of pediatric burn nursing.

**Funding for the study:**

N/A.

